# Impact of age on reperfusion success and long-term prognosis in ST-segment elevation myocardial infarction – A cardiac magnetic resonance imaging study

**DOI:** 10.1016/j.ijcha.2021.100731

**Published:** 2021-03-02

**Authors:** Divan Gabriel Topal, Kiril Aleksov Ahtarovski, Jacob Lønborg, Dan Høfsten, Lars Nepper-Christensen, Kasper Kyhl, Mikkel Schoos, Adam Ali Ghotbi, Christoffer Göransson, Litten Bertelsen, Lene Holmvang, Steffen Helqvist, Frants Pedersen, Renate Schnabel, Lars Køber, Henning Kelbæk, Niels Vejlstrup, Thomas Engstrøm, Peter Clemmensen

**Affiliations:** a Department of Cardiology, Rigshospitalet, Copenhagen University Hospital, Denmark; b Department of Cardiology, Zealand University Hospital, Denmark; c Department of Cardiology, Lund University Hospital, Lund, Sweden; d Department of Cardiology, University Heart Center Hamburg, Universitätsklinikum Hamburg-Eppendorf, Hamburg, Germany; e Department of Medicine, Nykøbing F Hospital, Nykøbing F, Institute for Regional Research, University of Southern Denmark, Odense, Denmark

**Keywords:** Collaterals, Collateral coronary circulation, CMR, Cardiac magnetic resonance, DANAMI-3, The Third Danish study on Acute Myocardial Infarction, ECG, Electrocardiogram, LVEF, Left ventricular ejection fraction, MVO, Microvascular obstruction, PCI, Percutaneous coronary intervention, STEMI, ST-segment elevation myocardial infarction, TIMI, Thrombolysis in myocardial infarction, ST-segment elevation myocardial infarction, Magnetic resonance imaging, Percutaneous coronary intervention, Age

## Abstract

**Background:**

Coronary collateral circulation and conditioning from remote ischemic coronary territories may protect culprit myocardium in the elderly, and younger STEMI patients could suffer from larger infarcts. We evaluated the impact of age on myocardial salvage and long-term prognosis in a contemporary STEMI cohort.

**Methods:**

Of 1603 included STEMI patients 807 underwent cardiac magnetic resonance. To assess the impact of age on infarct size and left ventricular ejection fraction (LVEF) as well as the composite endpoint of death and re-hospitalization for heart failure we stratified the patients by an age cut-off of 60 years.

**Results:**

Younger STEMI patients had smaller final infarcts (10% vs. 12%, P = 0.012) and higher final LVEF (60% vs. 58%, P = 0.042). After adjusting for multiple potential confounders age did not remain significantly associated with infarct size and LVEF. During 4-year follow-up, the composite endpoint occurred less often in the young (3.2% vs. 17.2%; P < 0.001) with a univariate hazard ratio of 5.77 (95% CI, 3.75–8.89; p < 0.001). Event estimates of 4 subgroups (young vs. elderly and infarct size beyond vs. below median) showed a gradual increase in the occurrence of the composite endpoint depending on both age and acute infarct size (log-rank p < 0.001).

**Conclusion:**

Having a STEMI after entering the seventh decade of life more than quadrupled the risk of future death or re-hospitalization for heart failure. Risk of death and re-hospitalization depended on both advanced age and infarct size, albeit no substantial difference was found in infarct size, LVEF and salvage potential between younger and elderly patients with STEMI.

## 1 Introduction

Although acute coronary syndrome is mainly attributed to the elderly, younger adults are also frequently affected [1]. In fact, the prevalence of ST-segment elevation myocardial infarction (STEMI) among younger patients has increased over the last decade, and accounts for almost 50% of all STEMI patients [1], [2], [3], [4]. This might be ascribed to an increase in risk factors, such as, hypertension [5], smoking [1], obesity [1], [4] and diabetes [6] among younger individuals, potentially leading to an increased STEMI incidence in the middle aged patients [7], [8].

Advanced age is associated with increased mortality in STEMI patients, however, younger patients still have a considerable mortality [9], [10]. Nevertheless, the mortality in patients with acute myocardial infarction increases exponentially with increasing age with a 5 times higher mortality in octogenarians [11]. Young patients are more often coronary disease naïve and less likely to present with multivessel disease [3], [12]. Multivessel disease increases the possibility of pre-infarction angina pectoris and hence preconditioning [13] that could be protective by mitigating reperfusion injury in the setting of STEMI. Elderly patients with STEMI are also known to have a better developed collateral coronary circulation (collaterals) [14], [15], and while it has been shown that pre-infarction angina relates to reduced infarct size and increased myocardial salvage [16], the data on the protective role of collaterals are conflicting [16], [17], [18], [19], [20], [21], [22]. To our knowledge the influence of age on myocardial salvage has never been evaluated. We hypothesized, that young STEMI patients, would have less salvage of jeopardized myocardium and larger infarct size in the setting of STEMI. Cardiac magnetic resonance (CMR) can measure area at risk [23], [24], [25], infarct size [25], [26], myocardial salvage index [23], [25] and microvascular obstruction (MVO) [27], [28] in STEMI patients. Additionally, myocardial salvage index and infarct size are strong predictors for outcome, and myocardial salvage index is assumed to be a robust surrogate for reperfusion success [29]. There exist no data on the association between young STEMI patients and myocardial salvage index and infarct size after primary percutaneous coronary intervention (PCI). The aim of the current study is therefore to evaluate the impact of age on reperfusion success as well as long-term prognosis in a contemporary STEMI cohort.

## 2 Methods

### 2.1 Study design

The present study is a substudy of the nationwide randomized multicenter DANAMI-3 (The Third Danish Study of Optimal Acute Treatment of Patients With ST- Segment–Elevation Myocardial Infarction) trial which is previously described in detail [30]. STEMI was defined as angina pectoris in conjunction with either ST elevation ≥ 0.1 mV in at least 2 contiguous leads in I, II, III, aVF, aVL, V4 through V6 or ST elevation ≥ 0.2 mV in at least 2 contiguous leads in V1 through V3 or presence of newly developed left bundle branch block.

As CMR was performed solely at Rigshospitalet, Copenhagen University Hospital, Denmark, the present study cohort is limited to this center. Exclusion criteria for CMR were potential pregnancy, known contrast allergy, severely impaired kidney function, atrial fibrillation/flutter or pacemaker or cerebral/cochlear implants.

Patients were included after written informed consent and the national ethical committee approved the study. The study was performed in accordance with the Helsinki declaration. The DANAMI-3-trial program was registered on www.clinicaltrials.gov under NCT01435408 (DANAMI 3-iPOST and DANAMI 3-DEFER) and NCT01960933 (DANAMI 3-PRIMULTI).

## 3 Study population and clinical outcome

In this DANAMI-3-trial substudy, we evaluated the impact of age on patients with STEMI with available CMR (n = 807) for imaging endpoints and the entire population included at Rigshospitalet (n = 1603) for clinical outcome. For the status “elderly” no universally acknowledged age cut-off exists but World Health Organisation suggests 60 to 65 in developed countries [31]. We attempted to match group sizes and chose to stratify by a 60 years age cut-off [31]. The primary clinical endpoint was a composite of all-cause mortality and re-hospitalization for heart failure, and secondarily we also reported evaluations of individual components. Events were collected from national registries and validated by reviewing hospital records. Heart failure was defined as prolongation of the index hospitalization due to worsening of heart failure or later hospitalization with heart failure requiring treatment. An independent clinical event committee adjudicated all events.

## 4 Cardiac magnetic resonance imaging

CMR was performed after primary PCI both during index admission (CMR index) and after 3 months (CMR follow-up). Both index and follow-up CMR examinations were performed on a 1.5 Tesla scanner (Siemens, Erlangen, Germany) using a 6-channel body array coil. Scout images as well as 2-, 3- and 4-chamber images were used to setup the short axis image plane. CMR index scan assessed area at risk, acute infarct size, MVO and left ventricular (LV) ejection fraction (EF). CMR follow-up scan assessed final infarct size and final LVEF. Area at risk was assessed using a T2-weighted short tau inversion-recovery sequence. Acute and final infarct size were assessed using delayed contrast enhanced electrocardiogram-triggered inversion-recovery imaging after intravenous injection of 0.1 mmol/kg body weight gadolinium-based contrast (Gadovist; Bayer Schering, Berlin, Germany). The inversion time was continuously adjusted to null the signal from the normal myocardium. Acute and follow-up LVEF were derived from steady state free precession cine imaging. All images were obtained in the short-axis plane with 8 mm slice thickness and 0 mm slice gap, covering the entire LV.

## 5 Image analysis

CVI42 (Circle Cardiovascular Imaging Inc, Calgary, Canada) was used for all quantitative analyses and performed by a reader blinded to all clinical and paraclinical data. A second blinded reader reviewed all analyses, and any relevant discrepancy was discussed and if necessary, adjusted until consensus was reached. On T2-weighted images area at risk was defined as hyperenhanced myocardium at a threshold of remote myocardium mean signal intensity ≥ 2 standard deviations (SD). Infarct size was identified as delayed gadolinium enhanced area with a threshold of remote myocardium mean signal intensity ≥ 5 SD [32]. Area at risk and infarct sizes are expressed as percentage of LV mass. Diffuse hyperintensive myocardial areas outside the area of the culprit lesion were excluded from the analysis of area at risk and infarct size. In addition, hypointensive areas within the area at risk were regarded as a part of the area at risk [24]. Myocardial salvage index was calculated as: (area at risk – infarct size)/area at risk [33]. MVO was assessed on CMR index as hypointensive areas within the infarct core on the delayed gadolinium enhanced images. LVEF, LV mass and LV volumes were measured on both examinations including papillary muscles as part of the LV volume.

### 5.1 Statistical analysis

Quantitative variables were tested for normality (Shapiro-Wilks test) described as mean with SD or median with interquartile range (IQR) and compared using student’s *t* test or Wilcoxon rank-sum test as appropriate. Qualitative variables were summarized by counts and percentages and compared with the chi-square test. A population division was made with age of 60 years as cut-off. Differences in time to event distributions were assessed by means of the log-rank test, and the Kaplan-Meier method was used to display event free survival probabilities. Clinical outcomes were examined with time-to-first-event analysis. We used Cox proportional hazards regression models to calculate hazard ratios with 95% confidence intervals (CI) and adjusted for variables that were significantly different between the groups, as well as potential confounders: multivessel disease; thrombolysis in myocardial infarction (TIMI) flow 0/1 pre-PCI; Killip class at admission; anterior STEMI location; time from symptom onset to balloon; diabetes; hypertension; family history of coronary artery disease; current smoking; troponin T peak value and gender. The assumption for the proportional hazards was found tenable by exploration of log (-log survival) curves and by testing the interaction term of each covariate with survival time. With linear regression we evaluated the association between age and infarct size as well as age and LVEF. In the linear regression models we adjusted for both risk-factors (diabetes; hypertension; family history of cardiovascular disease; current smoking; hyperlipidemia; gender; prior myocardial infarction) and outcome-predictors (anterior STEMI location; TIMI 0/1 pre-PCI; time from symptom onset to balloon). Using analysis of covariance, we tested for interaction between age and treatment (postconditioning, deferred stenting or multivessel revascularization) on the effect on acute infarct size. A two-sided probability value of 0.05 was the threshold for statistical significance. All statistical analyses were performed with SPSS software version 25.0 (SPSS Inc, Chicago, IL).

## 6 Results

**Fig. 1:**
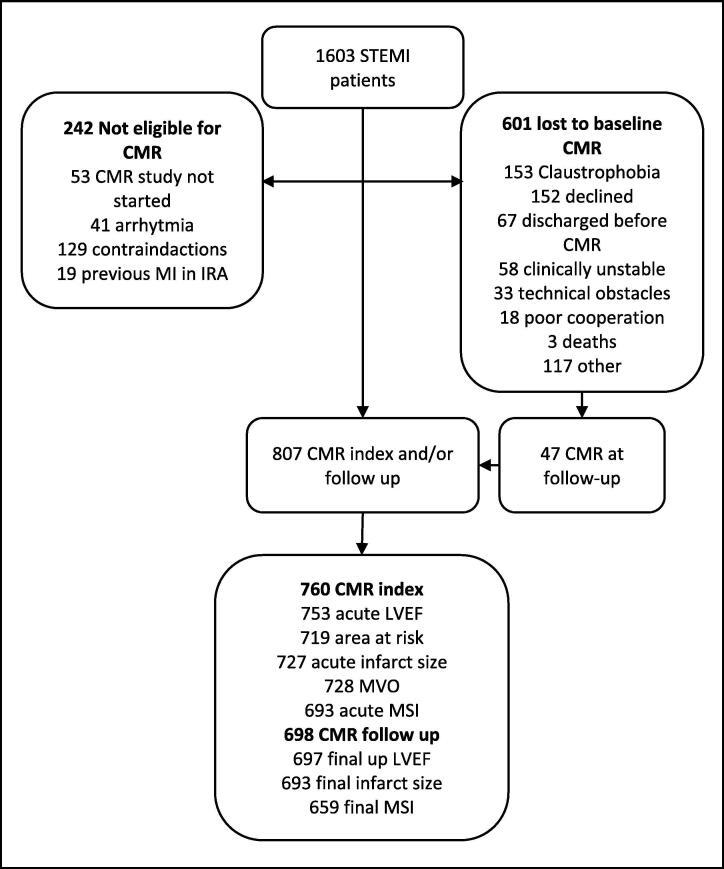
Trial profile. CMR, cardiac magnetic resonance; Follow up, 90 days after discharge; Index, index admission after percutaneous coronary intervention; IRA, infarct related artery; MI, myocardial infarction; MSI, myocardial salvage index; MVO, microvascular obstruction.

A total of 1603 STEMI patients were included from the DANAMI-3 trial at Rigshospitalet. 753 patients were under 60 years of age (47%) and 850 patients were 60 years or above (53%). Fig. 1 shows the trial profile. The median time from primary PCI to CMR index was 1 day (interquartile range, 1–1) and to CMR follow-up 91 days (interquartile range, 89–96). Baseline clinical and angiographic data for the total population and the CMR population are summarized in Table 1. The young patients had higher body mass index; had a lower prevalence of hypertension; were more likely to be smokers; were more likely to have a family history of cardiovascular disease and were more often male. Moreover, the young had shorter time from symptom onset to wire-crossing, more often presented with lower Killip class and had lower troponin-T peak values. Angiography revealed a lower percentage of multivessel disease in the young, and PCI more often resulted with TIMI-flow 3. In general, there were few differences in baseline data between the CMR-group and the total population. However, in the total population the younger patients more often were discharged with beta-blockers.

**Table 1:** Clinical and angiographic characteristics.

	All patients			CMR population		
	<60 years (n = 753) ≥ 60 years (n = 850) P			<60 years (n = 455) ≥ 60 years (n = 352) P		
Age, years	51 (6)	71 (7)	<0.001	51 (6)	69 (6)	<0.001
Male	613 (81%)	612 (72%)	<0.001	377 (83%)	264 (75%)	0.006
BMI	27 (25–29)	26 (24–29)	<0.001	27 (25–29)	26 (24–29)	0.001
**Risk factors**						
Diabetes	76 (10%)	81 (10%)	0.70	36 (8%)	31 (9%)	0.65
Hypertension	234 (31%)	422 (50%)	<0.001	130 (29%)	152 (43%)	<0.001
Hyperlipidemia	276 (37%)	299 (35%)	0.54	156 (34%)	131 (37%)	0.39
Current smoking	516 (69%)	316 (37%)	<0.001	312 (67%)	130 (37%)	<0.001
Family history of CAD	407 (54%)	323 (38%)	<<0.001	248 (55%)	148 (42%)	< 0.001
Previous MI	42 (6%)	60 (7%)	0.23	16 (4%)	17 (5%)	0.35
Previous PCI	47 (6%)	48 (6%)	0.62	19 (4%)	17 (5%)	0.66
Anterior STEMI	309 (41%)	366 (43%)	0.30	177 (39%)	157 (45%)	0.10
Heart rate, beats per min*	71 (59–84)	71 (59–84)	0.99	70 (59–84)	71 (59–84)	0.84
Systolic BP, mmHg*	131 (116–148)	135 (120–154)	0.23	131 (116–149)	135 (120–154)	0.062
Diastolic BP, mmHg*	80 (14)	77 (15)	0.015	80 (14)	76 (15)	0.005
Symptom to balloon, min	169 (124–267)	182 (139–271)	0.004	170 (124–271)	181 (139–277)	0.019
ECG to first wire, min	86 (68–115)	84 (71–110)	0.038	85 (69–114)	85 (71–113)	0.51
Peak Troponin-T, ng/L	2610 (954–5360)	3290 (1455–6038)	<0.001	2825 (1052–5540)	3360 (1620–6110)	<0.001
Killip class ≥ 2 **	19 (3%)	97 (11%)	<0.001	4 (1%)	26 (7%)	<0.001
**Culprit lesion**			0.15			0.10
Left Main	0	2 (0.2%)		0	1 (0.3%)	
LAD	305 (40%)	362 (43%)		176 (39%)	158 (45%)	
RCA	329 (44%)	372 (44%)		202 (44%)	153 (43%)	
LCX	119 (16%)	114 (13%)		77 (17%)	40 (11%)	
TIMI flow 0/1 pre-PCI	445 (59%)	516 (61%)	0.49	260 (57%)	223 (63%)	0.075
Multivessel disease	256 (34%)	391 (46%)	<0.001	153 (34%)	179 (51%)	<0.001
TIMI flow 3 post-PCI	731 (97%)	804 (95%)	0.014	446 (98%)	336 (96%)	0.037
**Medication at discharge**						
Aspirin	744 (99%)	826 (97%)	0.022	452 (99%)	342 (97%)	0.015
ADP inhibitor	745 (99%)	833 (98%)	0.24	454 (99,8%)	348 (99%)	0.20
Beta-blocker	705 (94%)	753 (89%)	<0.001	424 (93%)	322 (91%)	0.36
ACEI / ARB	285 (38%)	405 (48%)	<0.001	169 (37%)	165 (47%)	0.005

Data are shown as mean (±SD), median (interquartile range) or numbers (%). Probabilities are for categorical variables derived from x^2^ and for continuous variables from Student’s *t*-test or Wilcoxon rank-sum test as appropriate.

ACEI/ARB, angiotensin converting enzyme inhibitor/angiotensin-II receptor blocker; ADP, adenosine diphosphate receptor; BMI, body mass index; CAD, coronary artery disease; CMR, cardiac magnetic resonance; MI, myocardial infarction; PCI, percutaneous coronary intervention; STEMI, ST-elevation myocardial infarction.*, at admission; **, during index hospitalization.

## 7 Age in relation to long-term adverse outcome

**Fig. 2:**
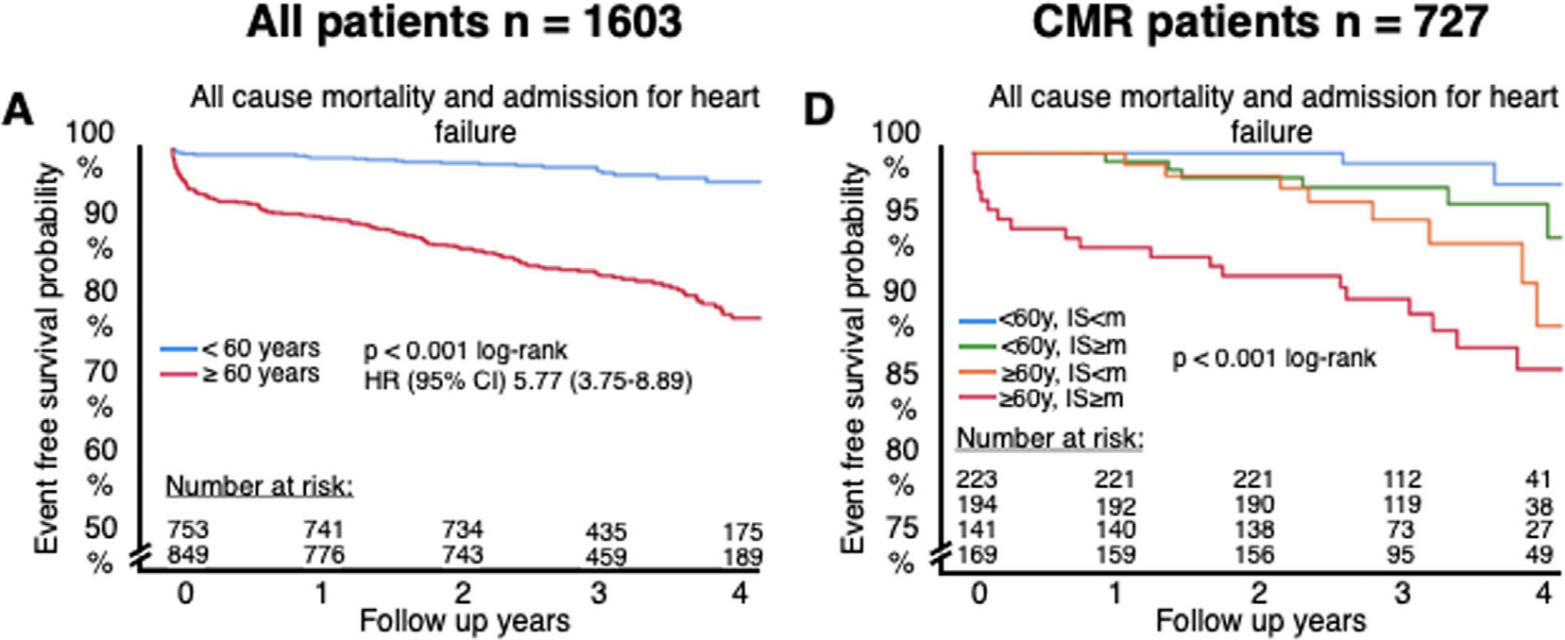
Kaplan Meier curves of endpoints stratified by age < 60 years vs. ≥ 60 years. A shows a curve for the total cohort and B for the CMR cohort further stratified by median infarct size. The shown hazard ratios are from univariate cox regression. CI, confidence interval; CMR, cardiac magnetic resonance; HR, hazard ratio; IS, infarct size; m, median; y, years.

The median follow-up time was 40 months (IQR, 30-48). As illustrated in Fig. 2A the composite endpoint (all-cause mortality and re-hospitalization for heart failure) occurred less often in the young (young patients, 24 [3.2%] versus elderly patients, 146 [17.2%]; p < 0.001), with a univariate hazard ratio for the composite endpoint of 5.77 (95% CI, 3.75–8.89; p < 0.001; Fig. 2A). Thus, entering the seventh decade of life more than quadrupled the risk of future death or re-hospitalization for heart failure. We also observed differences between young and elderly for each components of the composite endpoint (Fig. 2B and C); univariate hazard ratios for all-cause mortality and re-hospitalization for heart failure were 6.14 (95% CI, 3.63–10.38; p < 0.001) and 4.19 (95% CI, 2.26–7.54; p < 0.001), respectively. Similar findings were observed in the CMR cohort (not displayed in figure); composite endpoint (hazard ratio (HR), 4.23 [95% CI, 2.15–8.30; p < 0.001]), all-cause mortality (HR, 3.84 [95% CI, 1.73–8.56; p = 0.001]) and heart failure (HR, 4.81 [95% CI, 1.60–14.50; p = 0.005]).

## 8 Age in relation to infarct size, myocardial salvage and LVEF

CMR examinations were available in 807 patients (50.4%; Table 1) during index admission, at follow-up or both (CMR data are shown in Table 2). The CMR variables were discretely different among young and elderly STEMI patients: Young STEMI patients had a smaller acute and final infarct size, higher LVEF at both index and follow-up CMR as well as a smaller area at risk. We found no difference in MVO and myocardial salvage index. In adjusted linear regression analysis, there was no significant association between age and acute infarct size (β = 1.60; p = 0.06; Table 3) or final LVEF (β = −1.14; p = 0.13; Table 3). Age and treatment as per randomization (postconditioning, deferred stenting or multivessel revascularization) showed no interaction with CMR variables.

**Table 2:** Cardiac magnetic resonance data.

	<60 years		≥60 years		Relative difference	
	n		n			P
**CMR index**						
LVEF (%)	430	52 (45–58)	323	51 (43–57)	2%	0.14
Area at risk (%LV)	416	31 (24–38)	303	32 (25–40)	3%	0.034
Acute infarct size (%LV)	417	14 (7–22)	310	17 (8–25)	21%	0.005
Acute salvage index	405	0.49 (0.36–0.71)	288	0.48 (0.30–0.68)	2%	0.016
MVO (%LV)	418	0.58 (0–2.4)	310	0.71 (0–3.4)	22%	0.17
MVO present	418	200 (48%)	310	163 (53%)	10%	0.14
**CMR follow-up**						
LVEF (%)	409	60 (53–64)	288	58 (51–64)	3%	0.042
Infarct size (%LV)	406	10 (4–18)	287	12 (7–20)	20%	0.012
Final salvage index	372	0.69 (0.55–0.81)	287	0.65 (0.50–0.77)	6%	0.07

Data are shown as median with interquartile range and counts with percent. Probabilities derived from Wilcoxon rank-sum test. CMR, cardiac magnetic resonance; LV, left ventricular; MVO, microvascular obstruction.

**Table 3:** Adjusted linear regression for the association between age and acute infarct size as well as age and final LVEF.

	Infarct size acute		LVEF final	
	β	P	β	P
Age ≥ 60 y	1.60	0.06	−1.14	0.13
Diabetes	1.71	0.24	−1.53	0.21
Hypertension	−0.51	0.55	−1.20	0.12
Family history of CAD	−1.26	0.11	0.52	0.46
Current smoking	1.07	0.20	−1.75	0.016
Hyperlipidemia	−1.21	0.16	0.39	0.61
Male	2.90	0.003	−2.62	0.002
Prior myocardial infarction	0.98	0.65	−6.52	<0.001
Anterior STEMI location	7.26	<0.001	−3.83	<0.001
Symptom onset to balloon	0.01	0.010	−0.004	0.09
TIMI-flow 0/1 pre-PCI	9.73	<0.001	−5.02	<0.001

CAD, cardiovascular disease; LVEF, left ventricular ejaction fraction; PCI, percutaneous coronary intervention; STEMI, ST-segment elevation myocardial infarction and TIMI, thrombolysis in myocardial infarction.

## 9 Age and infarct size in relation to long-term adverse outcome

With linear regression, we evaluated the importance of age, stratified by the aforementioned 60 years cut-off, and infarct size stratified by the median on outcomes using the Kaplan Meier method. The composite endpoint of all-cause mortality depended on both age and infarct size with increasing age and infarct size resulting in higher rates of death and heart failure re-hospitalization (log-rank p < 0.001]; Fig. 2B).

## 10 Discussion

In line with previous studies we observed that nearly half of the present STEMI population was under 60 years of age. Younger patients with STEMI had a lower all-cause mortality and heart failure at admission – in accordance with previous findings –[9], [10] which on the other hand was dependent on infarct size even in the elderly who a priori had a poor prognosis. Male sex, anterior STEMI, symptom to balloon and pre-PCI TIMI 0/1 flow were the strongest independent predictors of (larger) infarct size. However, even though we showed significant smaller infarcts, higher LVEF and a numerical better salvage potential in younger STEMI patients, these differences were minor and of no clinical relevance.

The main focus in the present paper was to highlight the impact of age on myocardial salvage in patients with STEMI, and although the impact of age on prognosis was evaluated thoroughly before we still provide new data as mentioned above. Previous studies, firstly, either used extremely high or low age cut-offs compared to that recommended by the World Health Organization; Secondly, were somewhat antedated by not using contemporary STEMI protocols including prehospital treatments and loading with unfractionated heparin and P2Y12 inhibitor; Thirdly, included patients with previous myocardial infarction. Despite this we found a similar association between age and prognosis. Moreover, in contrast to another study we showed that prognosis is depended on both age and infarct size [34]. However, the study had a considerable number of patients with previous myocardial infarction and mixed assessment of infarct size by CMR and single photon emission computer tomography. These differences may explain the different results. Our initial assumptions that the elderly present with more risk factors, higher burden of previous interventions and infarctions was not entirely confirmed. In fact, only hypertension and multivessel disease was more prevalent among the elderly, and the higher number of completely occluded vessels on the initial angiogram and significantly lower TIMI flow post intervention were suggestive of a more complex coronary artery disease. The time from symptom to balloon is of paramount importance in STEMI patients, and our study suggests that the system response and/or alarming is swift in younger patients. Previous studies showed similar acute occlusion and reperfusion success rates in young patients [35]. TIMI 0/1 pre-PCI is a known indicator of increasing myocardial infarct size and adverse prognosis, and a lower rate of TIMI 3 post-PCI (successful PCI) is associated with detrimental consequences [9], [35]. These observations were highlighted in our study showing that symptom to balloon was independently associated with acute infarct size whereas age was not. The seemingly more timely treatment in the young cannot be explained by this study – possibly, high-risk characteristics such as the higher body mass index, smoking and family history of cardiovascular disease led to a higher awareness. Another factor potentially contributing to the good prognosis in the young patients in the DANAMI 3 trial was the larger proportion of young males who are known to have better prognosis than females [36].

Although collaterals were not assessed, these were unlikely to impact the result. If anything, one would assume that the elderly with documented more multivessel disease would have some protection from well-developed collateral circulation [14], [15]. Yet no substantial difference in infarct size and salvage potential was found between younger and elderly patients with STEMI. Whether advancing age in itself directly dictates the degree of collaterals remains debatable [18], [20]. Many strategies have aimed to increase reperfusion success and thereby mitigate the consequences of STEMI but from the current data, reducing time from symptom onset to revascularization seems pivotal as does cardiovascular risk factor control.

## 11 Study limitations

As observed in several acute studies CMR is not always feasible, and also in the DANAMI-3 study was not available in a considerable proportion, potentially introducing selection bias. We therefore compared the CMR subset with the entire cohort and found similar results. Furthermore, among the elderly the mean age was 2 years less in the CMR cohort; we speculate that inclusion of more old and fragile patients in the CMR cohort would have amplified the minor differences in infarct size. Unfortunately, the DANAMI-3 trial did not include an angiographic core laboratory for the assessment of collateral circulation to the culprit area, so the possible impact on infarct size could not be taken into account. CMR is associated with limitations in assessment of area at risk and infarct size which might have influenced on the real association between age and the current infarct parameters. Assessment of area at risk before day 3–5 results in underestimation of area at risk and assessment of acute infarct size before day 2–5 results in overestimation [37], [38]. It is yet a strength that all patients had the index CMR performed at the same day post-PCI. This equalizes the underestimation and leave out any influence from the underestimation. Importantly, assessment of CMR within 2 days after primary PCI gives the optimal environment to assess MVO accurately since MVO is stable in this interval [37], [39]. Current manuscript also provides with data on final infarct size from the follow-up CMR which was obtained in accordance with the recommendation [40]. Similarly revascularization itself might have disturbed the real association as well. Albeit the interobserver variability for assessment of area at risk and infarct size was well-satisfiable [41]. Finally, using another cut-off for age may have given different results.

## 12 Conclusion

Having a STEMI after entering the seventh decade of life more than quadrupled the risk of future death or re-hospitalization for heart failure, highlighting, based on our results, the need for awareness among all age groups regarding symptoms of a heart attack and the urgency to call the ambulance. Moreover, our data showed that the risk of death and re-hospitalization for heart failure depended on both advanced age as well as infarct size, albeit no substantial difference was found in infarct size, LVEF and salvage potential between younger and elderly patients with STEMI.

## Funding

The Danish Agency for Science, Technology and Innovation, Rigshospitalet Research Pool, the Danish Council for Strategic Research (Eastern Denmark Initiative to Improve Revascularization Strategies [EDITORS], grant 09-066994).

## CRediT authorship contribution statement

**Divan Gabriel Topal:** Data curation, Formal analysis, Funding acquisition, Investigation, Methodology, Project administration, Resources, Supervision, Validation, Visualization, Writing - original draft, Writing - review & editing. **Kiril Aleksov Ahtarovski:** Data curation, Formal analysis, Investigation, Methodology, Project administration, Resources, Supervision, Validation, Visualization, Writing - original draft, Writing - review & editing. **Jacob Lønborg:** Funding acquisition, Investigation, Methodology, Validation, Visualization, Writing - review & editing. **Dan Høfsten:** Investigation, Methodology, Resources, Visualization, Writing - review & editing. **Lars Nepper-Christensen:** Investigation, Methodology, Resources, Visualization, Writing - review & editing. **Kasper Kyhl:** Investigation, Methodology, Resources, Visualization, Writing - review & editing. **Mikkel Schoos:** Investigation, Methodology, Resources, Visualization, Writing - review & editing. **Ali Ghotbi:** Investigation, Methodology, Resources, Visualization, Writing - review & editing. **Christoffer Göransson:** Investigation, Methodology, Resources, Visualization, Writing - review & editing. **Litten Bertelsen:** Investigation, Methodology, Resources, Visualization, Writing - review & editing. **Lene Holmvang:** Investigation, Methodology, Resources, Visualization, Writing - review & editing. **Steffen Helqvist:** Investigation, Methodology, Resources, Visualization, Writing - review & editing. **Frants Pedersen:** Investigation, Methodology, Resources, Visualization, Writing - review & editing. **Renate Schnabel:** Investigation, Methodology, Resources, Visualization, Writing - review & editing. **Lars Køber:** Investigation, Methodology, Resources, Visualization, Writing - review & editing. **Henning Kelbæk:** Investigation, Methodology, Resources, Visualization, Writing - review & editing. **Niels Vejlstrup:** Investigation, Methodology, Resources, Visualization, Writing - review & editing. **Thomas Engstrøm:** Funding acquisition, Investigation, Methodology, Supervision, Validation, Visualization, Writing - review & editing. **Peter Clemmensen:** Investigation, Methodology, Supervision, Validation, Visualization, Writing - review & editing.

## Declaration of Competing Interest

The authors declare that they have no known competing financial interests or personal relationships that could have appeared to influence the work reported in this paper.
